# Q fever in cattle in some Egyptian Governorates: a preliminary study

**DOI:** 10.1186/1756-0500-7-881

**Published:** 2014-12-07

**Authors:** Mayada Gwida, Maged El-Ashker, Mohamed El-Diasty, Christina Engelhardt, Iahtasham Khan, Heinrich Neubauer

**Affiliations:** Department of Hygiene and Zoonoses, Faculty of Veterinary Medicine, Mansoura University, Mansoura, 35516 Egypt; Department of Internal Medicine and Infectious Diseases, Faculty of Veterinary Medicine, Mansoura University, Mansoura, 35516 Egypt; Animal Health Research Institute-Mansoura Provincial Laboratory, Mansoura, Egypt; Friedrich-Loeffler-Institut (FLI), Institute of Bacterial Infections and Zoonoses, 07743 Jena, Germany; University of Veterinary and Animal Sciences, Lahore, Pakistan

**Keywords:** *Coxiella burnetii*, Cattle, Egypt, ELISA

## Abstract

**Background:**

Q fever, caused by *Coxiella burnetii*, is a zoonosis with great public health significance and can cause financial losses to animal owners. The knowledge of the epidemiology of Q fever in Egypt is limited. Reports on this disease are scarce. In 2012 and 2013, we carried out this investigation to estimate the seroprevalence of antibodies to *Coxiella burnetii* in dairy cows of nine farms located in the lower Egyptian Governorates of Dakahlia, Damietta and Port Said. 1,194 blood sera were randomly collected from apparently healthy Holstein Friesian dairy cows. The collected sera were tested by ELISA for *Coxiella burnetii* antibodies.

**Results:**

All farms tested positive with seroprevalences ranging from 2.9 to 26.7% on farms with less than 200 animals and 9.8 to 20.0% in farms with more than 500 animals. 158 cows (13.2%) had anti-*Coxiella* antibodies.

**Conclusion:**

Q fever may be enzootic in the cattle herds investigated in Damietta, Port Said, and Dakahlia Governorates of the Nile delta in both smaller and larger herds. There is a need for further research on the impact of Q fever on both veterinary and public health. The results of this study should trigger more detailed epidemiological studies in ruminants as well as investigations into the etiology of atypical pneumonia and fever of unknown origin in humans.

## Background

Q fever, a zoonotic disease caused by *Coxiella burnetii*, was first described in Queensland, Australia in 1935, after an outbreak of “flu-like illness” among slaughter house workers [1]. Since that time, it has been reported in most countries throughout the world [2, 3]. Although *Coxiella burnetii* has been shown to infect a number of mammals, domestic ruminants especially sheep and goats are considered the main reservoir and source of human infection [4]. However, dairy cows may also be a source of human infection [2]. The main route of human infection is inhalation of contaminated aerosols, or dust containing bacteria shed by infected animals while milk may also play a role [5, 6]. The clinical manifestations of Q fever in humans are highly variable and range from asymptomatic or mild disease with complete recovery (which probably occurs in most cases) to a variety of clinical signs such as acute flu-like illness, pneumonia, hepatitis and chronic endocarditis leading to inaccurate and delayed diagnosis [2]. In animals, reproductive problems can occur including abortion, stillbirth, retained placenta, infertility, and weak newborns causing severe financial loss to the owner [7]. Asymptomatic and symptomatic animals may release *Coxiella burnetii* in large quantities at parturition. Shedding can also occur into feces and urine of domestic ruminants which may play role in maintaining and disseminating the agent to the environment. Coxiellae can persist in the environment for long periods and may spread for long distances via the wind [8]. *Coxiella burnetii* can also be excreted into the milk of infected animals for many months and even years due to local infection of the mammary gland [9].

The isolation of the pathogen is a reliable diagnostic method, but it remains time-consuming and hazardous [10, 11]. Since there is no characteristic clinical presentation for Q fever, epidemiological investigations mainly rely on serological tools. Hence, ELISA was found to be the method of choice for Q fever seroprevalence studies in man and animals [12]. Although Q fever in man and animals is a notifiable disease in many countries, it remains poorly reported and its surveillance is severely neglected. In Egypt, little information is available regarding Q fever and epidemiological studies are still scarce. Till now, no studies are available regarding the seroprevelance of *Coxiella burnetii* in dairy cattle in Dakahlia, Damietta, and Port Said Governorates, Egypt. We carried out this investigation to estimate the seroprevalence of *Coxiella burnetii* in the cattle populations in the respective regions.

## Methods

### The selection criteria and sampling protocol

**Figure 1:**
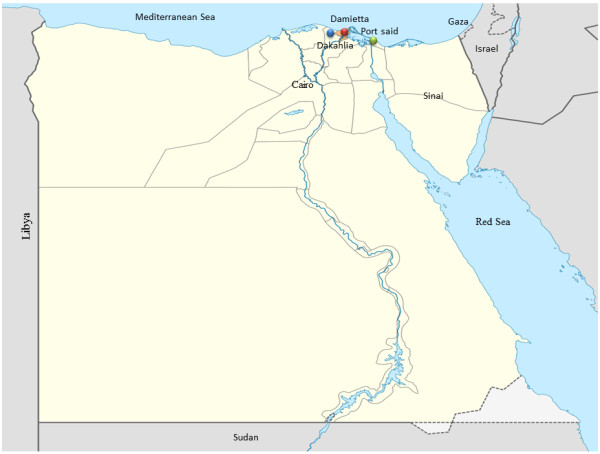
**Map of Egypt showing the location of study region in relation to the rest of Egypt (blue circle represents herds located in Dakahlia Governorate, red circle represents herds located in Damietta Governorate while green circle point out to a herd located in Port Said Governorate).**

The present study was conducted according to the principles of good clinical practice, and was approved by the Ethical Committee for Animal Experiments of Mansoura University. The present preliminary serological study included 1,194 apparently healthy Holstein Friesian dairy cows aged between 2 to 5 years on nine farms located in Dakahlia, Damietta and Port Said Governorates, Egypt (Table 1). These Governorates were chosen because convenient farms are located within a radius of 50, 85 and 135 km of Mansoura University (Figure 1). Five smaller holdings (less than 200 animals) and four holdings with more than 500 animals were included in the study. Ten ml of blood was collected from each animal through jugular venepuncture using plain tubes and needles. Each blood sample was labeled with the number of the respective animal. The collected blood samples were kept overnight at room temperature to allow blood clotting. On the next day, clear sera were collected and stored in cryotubes at -20°C until further examination. The samples were collected during routine brucellosis investigation within the context of the current brucellosis control program in the region and informed consent for the Q fever investigation was given by the owners.

**Table 1:** **Summary of cattle farms and ELISA positive samples from three lower Egyptian Governorates in the Nile Delta**

Number of farm	Location	Number of animals in the farm	Number of collected samples	Number of positives	%	Number of suspects	%
Farm 1	Damietta	1.200	50	10	20	1	2
Farm 2	Damietta	150	15	4	26.7	0	0
Farm 3	Dameitta	50	38	10	26.3	5	13.2
Farm 4	Damietta	600	336	50	14.9	16	4.8
Farm 5	Port Said	520	100	18	18	1	1
Farm 6	Dakahlia	170	167	26	15.6	7	4.2
Farm 7	Dakahlia	70	63	3	4.8	1	1.6
Farm 8	Dakahlia	70	69	2	2.9	1	1.5
Farm 9	Dakahlia	600	356	35	9.8	9	2.5
Total		3430	1194	158	13.2	41	3.75

### Serological assay

Serum samples were tested for Q fever antibodies (IgG) using ELISA (CHEKIT R; Idexx, Switzerland) according to the protocol of the manufacturer at a serum dilution of 1:400. The color development was measured as optical density (OD) with a spectrophotometer at 540 nm. The cutoff for positivity was determined by the %OD of the sample calculated as %OD = 100 × (OD value of test sample – OD value of negative control)/(OD value of positive control – OD value of the negative control). Samples with a %OD value less than 30% were considered negative for Q fever antibodies, those between 30% and 40% were considered suspect for Q fever antibodies, while those greater than 40% were considered positive.

## Results

All herds included in this preliminary serological study had animals with anti-*Coxiella* antibodies (n = 158; 13.2%). In small herds with less than 200 dairy cows, the prevalence ranged from 2.9 to 26.7% while in herds with more than 500 animals the prevalence was between 9.8-20.0% (Table 1). Seropositive herds were found in Damietta, Port Said and Dakahlia Governorates independent of the herd size (Table 1).

## Discussion

Q fever is a common zoonotic disease in most parts of the world with epidemiological data varying from country to another. In humans as well as in animals, the prevalence of Q fever is often unknown, and is usually underestimated since the presentation of the disease impedes its clinical diagnosis. In Egypt, few studies have been carried out in relation to this infection and still little is known about the epidemiology of *Coxiella burnetii* and its public health significance [13, 14]. Therefore, the need for a preliminary study to clarify the presence of Q fever in Lower Egypt in dairy cattle was evident.

In general, Q fever was not considered a potential problem in Egypt before 1995 as demonstrated by the virtual absence of reports on this disease before that time [15]. In 1995, Botros et al. described a seroprevalence of 25% in the risk group of cattle keepers. However, in the same study variable seroprevalence rates were reported; 20% among adult Egyptian blood donors in the Suez Canal area (n = 358); 16% in the Nile Valley (n = 501) and 10% in the Nile Delta (n = 427). On the other hand, in sheep and goats the seroprevalence rates were reported to be 22.5% and 16.5% in the North Sinai, respectively [13]. The recent finding of seropositive animals in Giza, Cairo and El-Fayum districts (7 of 54 animals examined) suggested that Q Fever may indeed be circulating in cattle herds in Egypt. In the present investigation, the seropositivity of *Coxiella burnetii* was lower than that reported from Nigeria, Sudan, Zimbabwe, Cameroon and Togo [16–20], but higher than those reported from Trinidad, Chad or Iran [3, 21, 22]. However, it should be noted that these studies differ in their design and laboratory and statistical analyses, thereby limiting their comparability with our results [21]. To better understand the epidemiology of *Coxiella burnetii* in the study area, further research will include the investigation of milk samples, uterine discharges and abortion material for the presence of the organism. These materials were not available in our study, although one of the ELISA positive serum samples from herd 1 was positive for *Coxiella burnetii* DNA corresponding to 9.5 genome equivalents by real time PCR. Our dairy cows were clinically healthy and no ticks, as potential reservoirs, were observed on the animals or in the housing areas upon examination. Generally, early acute Q fever infection is characterized by the presence of circulating *Coxiella burnetii* DNA in the absence of circulating antibodies (seronegative stage). Subsequently, IgM antibodies to phase II antigens (IgM-II) appear (seropositive stage) with circulating *Coxiella burnetii* DNA still present in most cases. Thereafter, phase II IgG (IgG-II), phase I IgM (IgM-I) and phase I IgG (IgG-I) antibodies appear with coinciding disappearance of circulating *Coxiella burnetii* DNA allowing for distinguishing time dependent serologic profiles [23]. Thus, it seems likely that the ELISA positive cattle were exposed to *Coxiella burnetii* in the past and some of these animals may have been chronically infected. These animals may be responsible for maintenance of the chain of infection. However, the ways of transmission still require further investigation.

A limitation of this study is that farms were selected by convenience sampling and are therefore not representative of the entire cattle population. However, this study is valuable in that it provides evidence for the circulation of *Coxiella burnetii* in the study area and people who deal with cattle may therefore be at risk to contract Q fever. Further epidemiological investigations are warranted. Given the lack of recent published data on Q fever in Egypt, the finding that all investigated herds included at least one seropositive animal is concerning. This conclusion is in line with a study in animal keepers which demonstrated that exposure was not uncommon [15]. In this regard, a recent interesting study dealing with the clinical spectrum of fever of unknown origin (FUO) among adult Egyptian patients admitted to Ain Shams University Hospitals has found that brucellosis and infective endocarditis were the most common causes of FUO (41.94%) followed by malignancies (30.11%) and autoimmune diseases (15.05%) while diagnosis was not reached in (12.9%) of patients [24]. Therefore, there is an urgent need for future studies on the impact of Q fever on both veterinary and public health.

## Conclusion

The results presented herein offer evidence that Q fever may be enzootic in cattle herds in Damietta, Port Said, and Dakahlia Governorates of the Nile delta region. In addition, the dimension of economic losses caused by abortion and mastitis has to be quantified. There is also an urgent need for future studies on the impact of Q fever on both veterinary and public health. The results of this study should trigger more detailed epidemiological studies in ruminants as well as investigation into the etiology of atypical pneumonia and fever of unknown origin in humans.
